# Nonprecious bifunctional electrocatalysts for oxygen electrode reactions

**DOI:** 10.3762/bjnano.17.90

**Published:** 2026-09-24

**Authors:** Shaheen Parveez Bhuyan, Bhrigu Kumar Pegu, Darshan Jyoti Gogoi, Pragya Moni Gogoi, Pankaj Bharali

**Affiliations:** 1 Department of Chemical Sciences, Tezpur University, Napaam 784028, Assam, India

**Keywords:** bifunctionality, electrocatalysis, non-precious electrocatalyst, oxygen evolution reaction, oxygen reduction reaction

## Abstract

The two most crucial reactions in energy storage and conversion systems are oxygen reduction reaction (ORR) and oxygen evolution reaction (OER). However, their sluggish kinetics arise the need for commercially feasible bifunctional electrocatalysts to assist the reaction by improving its kinetics at the same time ensuring durability and also taking cost into account. The fundamentals of the oxygen electrodes are discussed followed by various techniques adopted by researchers to tune the electronic structures of carbon- and transition metal-based nanostructures towards enhanced activity in ORR/OER. Emphasis is placed on correlating catalyst structure/composition with electrocatalytic properties, simultaneously highlighting the persistent challenges. Hence, the review provides an overview on the recent advances in the field of non-precious bifunctional electrocatalysts for oxygen electrode reactions, which will serve as a foundation for their application in various fields including metal–air batteries.

## Introduction

Traditional energy resources are becoming scarcer as societies and economies grow because of widespread consumption of fossil fuels. This overreliance on fossil fuels has caused severe environmental degradation resulted in glacier melting, depletion of the ozone layer, rising sea levels, and an increase in global temperatures. Hence addressing these challenges has become a critical priority in the modern era. To reduce energy constraints and environmental deterioration, the development of sustainable and environmentally benign energy conversion and storage technologies is essential. Regarding innovative technologies that lessen the carbon footprint, electrocatalysis has taken the forefront. Electrochemistry is crucial because it allows inter-conversion between electrical and chemical energy, providing surplus electrical energy, particularly from renewable sources to be stored in the form of chemical energy or utilized to drive non-spontaneous reactions. Conversely, energy shortages can occasionally be compensated by transforming stored chemical energy into electrical energy through natural electrochemical processes that take place in galvanic cells helping to balance energy supply and demand [1–3].

Recently, the improvement of effective and inexpensive energy tools has drawn a lot of research interest around efficient, low-cost, and green energy technologies. Water electrolyzers, fuel cells, organic solar cells, and metal–air batteries are regarded as promising, clean, and efficient for sources in the traditional energy sector due to their minimal environmental impact and higher energy transition efficacy. Using advanced sustainable electrochemical conversion technologies and catalytic principles reduces the dependence on fossil fuel-based energy sources [4].

## Review

### Fundamentals of oxygen electrode reactions

The electrocatalytic conversion of oxygen comprises oxygen reduction reaction (ORR) and oxygen evolution reaction (OER). ORR takes place at the cathode, whereas OER occurs at the anode. Both reactions proceed through multistep electron-transfer pathways, which ultimately govern the reaction kinetics. Notably, ORR and OER are four-electron reaction processes that result in intrinsically slow kinetics and higher overpotentials [5–6]. Pt/C is the standard catalyst for ORR, while Ir/Ru oxides have demonstrated exceptional stability and durability for OER. However, the high cost and limited availability of these precious noble-metal based catalysts restrict their large-scale practical applications. Pt-based catalysts have a strong ORR activity but poor OER performance due to the formation of surface Pt oxides that develop at high overpotentials and possess low electrical conductivity. In contrast, for OER, RuO_2_ and IrO_2_ show superior catalysis but are poor in ORR performance [1,7]. Therefore, it is essential to concentrate on creating a suitable bifunctional OER/ORR catalyst that replaces noble metal catalysts with inexpensive, abundant, and highly active non-noble metal catalysts. The most common approach of identifying a good bifunctional catalyst for ORR and OER is to measure its overpotential based on the same catalyst loading and at a specific current density. A promising bifunctional catalyst is indicated by a smaller overpotential at a specific rate. Additionally, a bifunctional catalyst that is both highly active and stable enough to cope with the challenging conditions faced during ORR and OER needs to be developed [6].

In recent years, transition metal (TM)-based and carbon-based materials appeared as promising candidates for bifunctional ORR/OER electrocatalysts. Their favourable catalytic properties, including higher activity, high surface area, selectivity, and tunable electronic structures, make them suitable for enhancing the electrochemical performance in energy storage and conversion applications. Transition metals, such as, Fe, Co, Mn, Ni, Cu, and Mo, and their compounds are highly desirable due to their cost-effectiveness, catalytic activity and compositional diversity. In comparison to noble metals like Pt, and Ir, TMs are more abundant and less expensive [8]. TM compounds can be classified into several categories based on their composition and structure, including metal–organic frameworks (MOFs) [9], alloys [10], hydroxides [11], dichalcogenides, phosphides, and nitrides [12–13]. Among these, transition metal alloys benefit from synergetic effects that can enhance performance via modifications in electronic structure and active sites. Furthermore, the catalytic activity of TM-based catalysts is quite comparable to that of noble metal-based catalysts. The performance of transition metal-based catalysts can be enhanced through doping or forming heterostructures. Surface chemical activity and charge transport characteristics can be modulated by doping metals and non-metals [14–15].

Strategies aimed at optimizing the intrinsic catalytic activity of non-precious metal electrocatalysts are fundamentally guided by the Sabatier principle. It states that the interaction between a catalyst surface and reaction intermediates must be perfectly balanced, that is, neither too strong nor too weak. A number of electronic structural factors such as d-band centre, *e*_g_ orbital occupancy, adsorption energies, density of states, and spin ordering have been identified as key descriptors in order to assess the bonding strength between reactants and active sites. These descriptors have emerged as valuable tools for understanding and predicting catalytic behaviour through quantitative structure–activity relationships. Among the descriptors, *e*_g_ occupancy and d-band centre are the most prevalent for predicting the activities of ORR catalysts; for OER catalysts, *e*_g_ occupancy and metal–oxygen covalency are most widely used [16]. In TM-based catalysts, the position of the d-band centre relative to the Fermi level influences the adsorption strength of oxygen-containing intermediates (*OH, *O, and *OOH), thereby affecting ORR and OER kinetics. Similarly, transition-metal cations have an *e*_g_ orbital filling close to unity, which often correlates with optimal oxygen intermediate adsorption and enhanced OER activity [17]. In addition, adsorption energies of key reaction intermediates serve as quantitative indicators of catalytic efficiency and can be used to establish volcano-type relationships between catalyst composition and activity [18]. Accordingly, a number of electronic structure engineering techniques have been set forward to maximize the intrinsic activity of a single catalytic site through generation of vacancies, heteroatom doping, and interface design [19].

Likewise, carbon-based materials have drawn great attention as electrocatalysts due to their cost-effectiveness, high surface area, superior electrical conductivity, economic viability, and strong synergistic interactions with other catalytic components. Carbon-based catalysts include carbon nanotubes (CNTs), graphene, and carbon black [18,20]. Graphene, a 2D single layer of sp^2^-bonded carbon atoms, and CNTs, known for their exceptional conductivity and surface area, have shown promising bifunctional activity towards both ORR and OER, even as metal-free catalysts in certain cases [21–22]. A typical approach for determining the superior bifunctional catalytic performance for ORR and OER is to measure the overpotential required to achieve a specific current density using the same catalyst loading for both ORR and OER. A lower overpotential at a given current density indicates superior bifunctional activity. Nanomaterials have distinctive properties that significantly enhance OER and ORR kinetics [15].

To date, several excellent review publications have summarized the progress of oxygen electrocatalysts, with emphasis on specific catalyst classes, important descriptors for catalytic activity, as well as synthetic strategies and practical applications [14,18,20–22]. In contrast, this review begins with a discussion of fundamental reaction mechanisms of ORR/OER, followed by discussion of various types of nanostructured catalysts like transition metal-based catalyst, carbon-based materials, their roles in improving ORR/OER performance, and developments in the design and synthesis of heterogeneous bifunctional electrocatalysts. Particular emphasis is placed on correlating catalyst composition and electronic structure with electrocatalytic performance through key activity descriptors of oxygen intermediates. Finally, the study explores current challenges and future directions in oxygen electrocatalysis.

### Reaction mechanisms of ORR and OER

The ORR operates at the cathode of fuel cells and metal–air batteries during discharge. The OER takes place while charging metal–air batteries or during water splitting [23–24]. The primary element of the oxygen electrode is the catalyst activation layer, where adsorbed oxygen participates in electrochemical processes that occur at the gas–liquid–solid three-phase boundary [25]. It involves multistep, slow proton-coupled electron-transfer processes in both ORR and OER, resulting in high overpotentials and low energy efficiency, underscoring the need for effective bifunctional electrocatalysts.

**ORR mechanism.** ORR represents one of the most vital processes studied in modern electrochemistry. It is either a two-electron process, which is linked to terminal oxygen adsorption and generates intermediate H_2_O_2_, or the complete transformation of molecular oxygen into water through a direct four-electron transfer process by bidentate oxygen adsorption [26–30]. The combination of rapid kinetics, effective electron transfer, and strong energy efficiency contributes to its appeal, with no reactant molecule loss and environmentally friendly reaction products. Four-electron reduction is ideal in the progress of distinct green energy strategies, for example, electrochemical energy conversion as well as storage [9]. In addition to producing corrosive products (H_2_O_2_), the indirect four-electron reaction and OH^−^ ions damage the catalyst and diminish the potential of the oxygen reduction electrode, hence decreasing battery efficiency. Therefore, the indirect four-electron reaction should be avoided as much as feasible [25].

**Figure 1:**
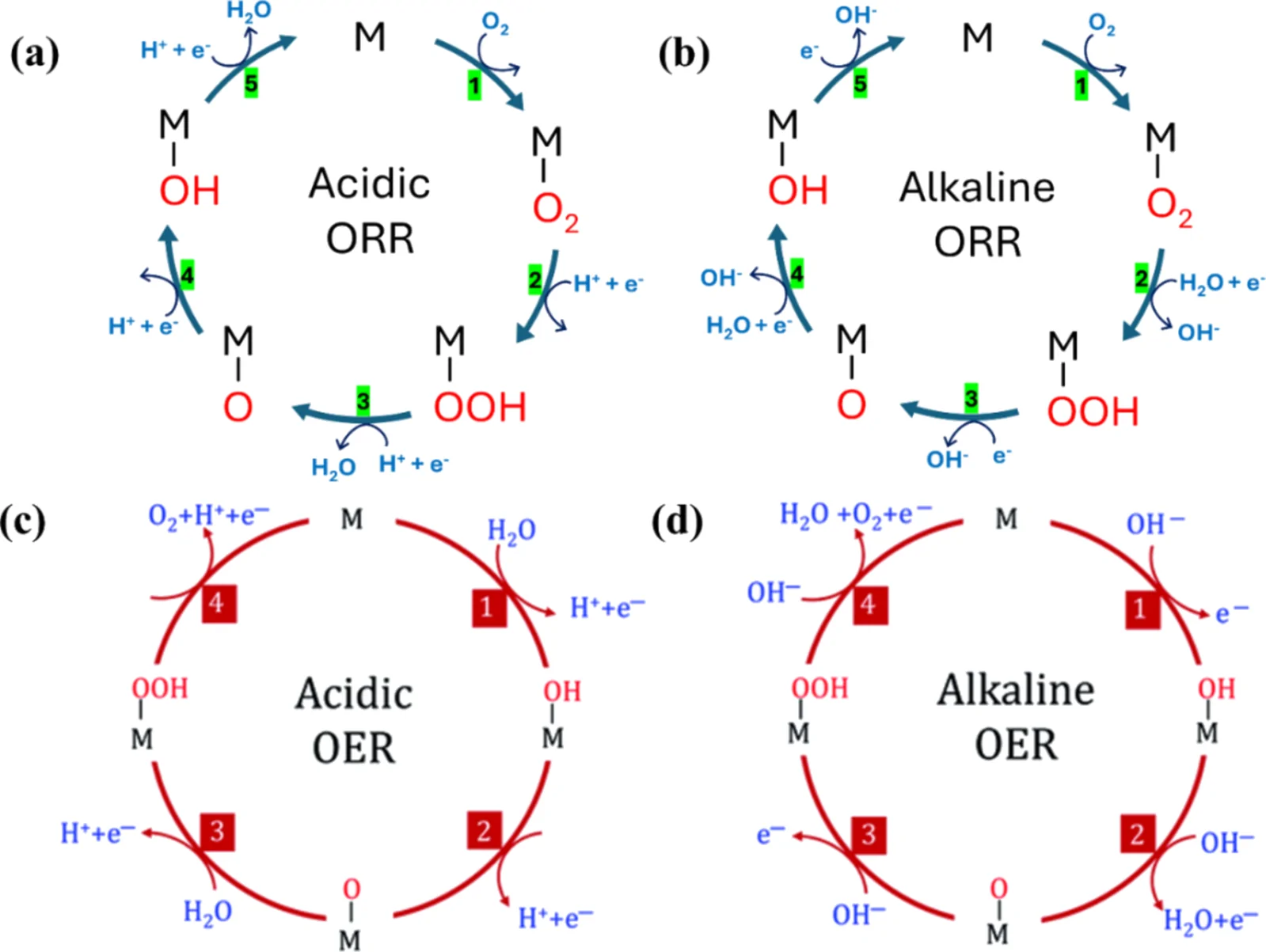
Schematic reaction mechanisms under (a) acidic and (b) alkaline conditions for ORR [35], and under (c) acidic and (d) alkaline conditions for OER [36] (Figure 1c and d was adapted from [36] © 2020 Z. Yan et al., published by Royal Society of Chemistry, distributed under the terms of the Creative Commons Attribution 3.0 Unported License, https://creativecommons.org/licenses/by/3.0/.)

In the direct four-electron process, the adsorption and activation of O_2_ molecules on the catalyst’s surface is a crucial stage. It proceeds through a series of surface or interfacial processes. Further, the reactions proceed via electron transfer, chemical bond breaking, molecular restructuring, and the creation of reaction products, which are ultimately discharged from the catalyst surface. Its activation is facilitated by stable O_2_ adsorption behaviour [31–32]. The processes of M–OH, M–O, and M–OOH adsorption and desorption are recognised as very significant in the reaction process, regardless of acidic or alkaline environments [33–34]. A detailed mechanistic route in both acidic and alkaline media is shown in Figure 1a,b and Table 1.

**Table 1:** Mechanistic pathways and thermodynamic parameters of ORR and OER under acidic and alkaline conditions [32–33].

Reaction pathway	Medium	Specific mechanistic route	Surface intermediates	Thermodynamic parameters (overall reaction)

ORR (4e^−^ route)	acidic	M + O_2_ → M–O_2_ M–O_2_ + H^+^ + e^−^ → M–OOH M–OOH + H^+^ + e^−^ → M–O + H_2_O M–O + H^+^ + e^−^ → M–OH M–OH + H^+^ + e^−^ → M + H_2_O overall reaction: O_2_ (g) + 4H^+^(aq) + 4e^−^ → 2H_2_O(liq)	M–O_2_ → M–OOH → M–O → M–OH	*E*° = 1.229 V vs RHE Δ*G*° = −4.92 eV
ORR (4e^−^ route)	basic	M + O_2_ → M–O_2_ M–O_2_ + H_2_O + e^−^ → M–OOH + OH^−^ M–OOH + e^−^ → M–O + OH^−^ M–O + H_2_O + e^−^ → M–OH + OH^−^ M–OH + e^−^ → M + OH^−^ overall reaction: O_2_ (g) + 2H_2_O (liq) + 4e^−^ → 4OH^−^ (aq)	M–O_2_ → M–OOH → M–O → M–OH	*E*° = 1.229 V vs RHE Δ*G*° = −4.92 eV
ORR (2e^−^ route)	acidic	M + O_2_ → M–O_2_ M–O_2_ + H^+^ + e^−^ → M–OOH M–OOH + H^+^ + e^−^ → M + H_2_O_2_ overall reaction: H_2_O_2_ (liq) + 2H^+^ (aq) + 2e^−^ → 2H_2_O (liq)	M–O_2_ → M–OOH	H_2_O_2_ formation (no Δ*G*° reported)
ORR (2e^−^ route)	basic	M + O_2_ → M–O_2_ M–O_2_ + H_2_O + e^−^ → M–OOH + OH^−^ M–OOH + e^−^ → M + OH_2_^−^ overall reaction: O_2_ (g) + H_2_O (liq) + 2e^−^ → HO_2_^−^ (aq) + OH^−^ (aq)	M–O_2_ → M–OOH	OH_2_^−^ formation (no Δ*G*° reported)
OER	acidic	H_2_O (liq) + M → M–OH + H^+^ + e^−^ M–OH → M–O + H^+^ + e^−^ H_2_O (liq) + M–O → M–OOH + H^+^ + e^−^ M–OOH → M + O_2_ (g) + H^+^ + e^−^ overall reaction: 2H_2_O (liq) → O_2_ + 4H^+^ + 4e^−^	M–OH→ M–O → M–OH	*E*° = 1.229 V vs RHE Δ*G*° = +4.92 eV
OER	basic	M + OH^−^ → M–OH + e^−^ M–OH + OH^−^ → M–O + H_2_O (liq) + e^−^ M–O + OH^−^ → M–OOH + e^−^ M–OOH + OH^−^ → M + O_2_ (g) + H_2_O (liq) + e^−^ overall reaction: 4OH^−^ → O_2_ (g) + 2H_2_O (liq) + 4e^−^	M–OH → M–O → M–OH	*E*° = 1.229 V vs RHE Δ*G*° = +4.92 eV

**OER mechanism.** Often regarded as the main obstacle in bifunctional oxygen electrodes, the OER has sluggish kinetics that constrains the capability to convert energy efficiently. Many studies over the past few years have focused on the development and understanding of OER. Numerous studies have been conducted utilizing density functional theory (DFT) simulations to examine the OER. Under both acidic and alkaline conditions, the commonly established OER process consists of four-electron/proton transfer steps [37]. During the OER mechanism, M–OH, M–O, and M–OOH species are adsorbed onto the crucial active site (M) of the electrocatalyst, which leads to the formation of O_2_ [38–39]. Under acidic conditions, H_2_O is dissociated to OH^−^ and gets adsorbed at the active site [6]. Under basic conditions, hydroxide ions first produce hydroxy intermediates (M–OH) by adhering to the electrocatalyst surface. After reacting with other OH^−^ ions, these intermediates create hydroperoxide species (M–OOH), which are then followed by the creation of O–O bonds [40]. A detailed mechanistic route in both acidic and alkaline media for OER is shown in Figure 1c,d.

The ORR and OER processes proceed via the continuous formation and transformation of intermediates at the catalytically active site. The catalytic activity and reaction energetics are largely determined by the adsorption behaviour of these intermediates. As a result, M–OH, M–O, and M–OOH adsorption free energies are commonly acknowledged as important indicators for assessing the intrinsic activity of ORR and OER electrocatalysts. In order to facilitate formation, conversion, and desorption of these intermediates during the reaction, an effective catalyst should have modest adsorption strengths. This concept is often demonstrated using activity volcano plots, where catalysts positioned around the volcano apex display adsorption energetics close to the optimum, thus providing enhanced electrocatalytic performance with reduced overpotentials [31,38–39]. Affordable and highly efficient bifunctional electrocatalysts having stable O_2_ and OH^−^ adsorption behaviour are currently needed. This review will elaborate on how carbon-based and non-precious transition metal-based electrocatalysts have been the popular candidates for such bifunctional oxygen electrodes.

### Bifunctional oxygen electrocatalysts

**Figure 2:**
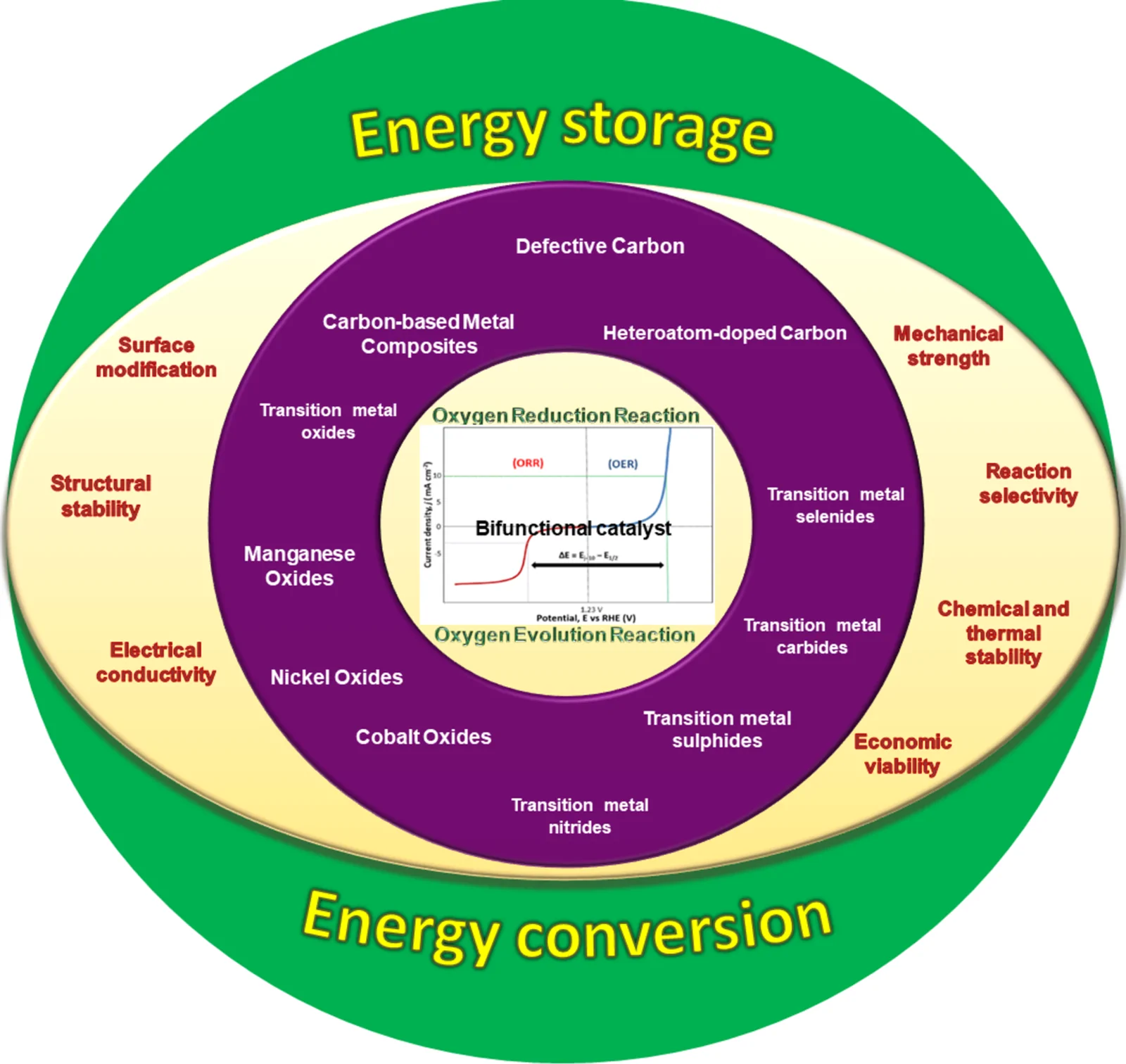
Overview of bifunctional electrocatalysts for oxygen electrode reactions in energy storage and conversion systems.

In recent decades, a variety of extremely efficient electrocatalysts made from non-precious materials have been designed and utilized for bifunctional ORR/OER reactions. Materials based on non-precious transition metals and carbons have demonstrated significant promise in electrochemical applications [41]. The increased surface area and exceptional electrically conductive nature of carbon-based materials help to increase ORR/OER activity. However, many carbon-based electrocatalysts face durability issues in the intense oxidative environment at high OER potentials, while transition metal-based electrocatalysts remain stable under such conditions [42]. Attaining a well-balanced performance that encompasses both ORR and OER capabilities is crucial for evaluating the catalytic effectiveness of a catalyst with bifunctionality towards its application in energy storage and energy conversion systems as displayed in Figure 2 [43].

The bifunctionality index (BI), that is, the potential difference (Δ*E* = *E_j_*_=10_ − *E*_1/2_), where E*_j_*_=10_ is for OER and the *E*_1/2_ for ORR, is a crucial performance metric (*E_j_*_=10_ indicates the potential at current density 10 mA·cm^−2^, and *E*_1/2_ indicates the half-wave potential). A lower potential for *E_j_*_=10_ or a more positive *E*_1/2_ means the catalyst possesses either a higher exchange current density; this means that the electrode surface has more active sites, better intrinsic conductivity, or faster charge-transfer kinetics, which allow the oxidation or the reduction to occur faster. Therefore, a smaller Δ*E* value signifies superior oxygen electrode activity and greater reversibility, highlighting the catalyst’s effectiveness [44]. The BI is derived from the simple calculation of *j(*η_OER_) and *j(*η_ORR_) from the current density (*j*) vs electrode potential (*E*) curve using voltammetry data [45]. While voltammetry tests provide a fast and straightforward experimental approach to determine the BI, it is important to recognize that discovering materials with outstanding bifunctional performance remains quite challenging. This difficulty largely stems from differences in the active sites involved in the OER and ORR, which are often altered by changes to the catalyst surface caused by the applied electrode potentials [46].

### Carbon-based materials

Currently, carbon-based materials are most widely used in the preparation of electrode materials because of their high availability, high conductivity, strong tolerance towards acid/alkaline condition, and low cost. Here, various aspects of the use of carbon-based materials as bifunctional electrocatalysts for ORR and OER are discussed.

**Defective carbon/defect engineering.** Carbon materials, like any other material, are not defect-free crystals. The defects are critical to the intrinsic properties of the carbon materials towards electrocatalysis. Hence, regulating such defects can ensure optimized OER and ORR catalytic activity, which is called defect engineering [47–49]. Intrinsic carbon defects include edges, vacancies, and topological defects [50]. These defects can affect the electrocatalytic activity by modifying the surface properties and electronic structure of the substrate [51]. Therefore, introducing intrinsic defects by defect engineering either modulates the p-orbital alignment, narrows the bandgap, or breaks the uniform π conjugation of the carbon framework accelerating the kinetics as well as creating active sites for the interaction of oxygen intermediates. This section will be discussing how intrinsic defect engineering can correlate to the understanding of electrocatalytic activity of a catalyst towards an electrochemical reaction.

Edges are the most common defects among the intrinsic carbon defects. Different electrochemical and thermodynamic properties are related to the edges depending on the states in which the edge and basal planes interact with each other [52]. A carbon atom at the edge with sp^2^ hybridization has a catalytic activity by a factor of two higher than a carbon atom in the bulk [53]. Tao et al. found that carboxylic groups at the defected edges, together with localised unpaired electrons facilitate intermolecular arrangements. These features work in tandem to effectively capture molecular oxygen, thereby improving the kinetics of the catalysis [54]. In a hexagonal carbon skeleton, there are two types of edges, armchair and zigzag edges [54]. Research revealed that due to the abundance of unpaired π electrons at the zigzag edge sites, the energy required to promote the formation of OOH^*^ from oxygen molecules is lower. However, a DFT calculation reported by Xia and co-workers also suggested that defects at the armchair edges of graphene showed lower overpotential than zigzag edge defects [55]. Holes or vacancies are another type of intrinsic defects in the basal plane of carbon. Vacancy defects occur when one to few carbon atoms are missing from the lattice, while hole defects involve missing of a large number of atoms. However, vacancy or hole defects are usually accompanied by edge defects. Dai and his group reported the synthesis of a graphitic surface with abundance of stereoscopic holes, which ensured high surface area and high pore volume, with abundant interfacial active surface area and good electron/electrolyte transfer enhancing the overall electrocatalytic activity of the catalyst towards ORR and OER [56].

Topological defects in carbon, also known as lattice imperfection, cause non-hexagonal structures like Stone–Wales (SW) defects in the carbon framework [57–58]. SW defects are prevalent topological defects in planar low-dimensional structures. Khan et al. developed a synthetic strategy of absorption–calcination, to fabricate SW defects to allow the incorporation of Ni and Fe into structure. It was found that the defect-rich carbon support enhanced the bifunctional activity of the catalyst and also provided control over agglomeration of Ni and Fe atoms [59].

**Heteroatom-doped carbon.** Hetero-doping the carbon skeleton with elements such as N, P, S, O, B, or F introduces a redistribution of electrons due to the difference in atomic size, bond length and also, electronegativity between the dopant and the carbon; this enables the catalyst to provide more active sites. Conceptually, doping nitrogen into a carbon-based material will cause the carbon to acquire positive charge by delocalisation, which will make such centres active sites for ORR [60]. Xia and co-workers established a volcano plot for the lower limit of overpotential of various p-block elements doped in graphene versus the descriptor Φ to correlate the binding energy with the ORR/OER activity of a metal-free heteroatom-doped carbon catalyst, where Φ is the dimensionless product of relative electron negativity (of the doping element relative to carbon) and electron affinity. According to the volcano plot, nitrogen was the most active dopant towards the ORR, whereas for OER, phosphorus exhibited the lowest overpotential [60]. Also, N-doped carbon is the most extensively studied heteroatom-doped carbon-based electrocatalyst because of the similar size of nitrogen atoms and carbon atoms with much higher electronegativity; less studies have been carried out for phosphorus due to its size and the difficulties in introducing it into the carbon matrix [61]. For example, Li and co-workers synthesized a wrinkled carbon nanosphere doped with nitrogen by a soft-template method. This improved the catalytic performance of both ORR and OER by providing a protective shell against agglomeration of M–Nx active sites and preventing alloy corrosion by accelerated surface layer reconstruction. The wrinkles on the nanospheres immobilized the active sites, thereby preventing them from agglomeration [62].

**Figure 3:**
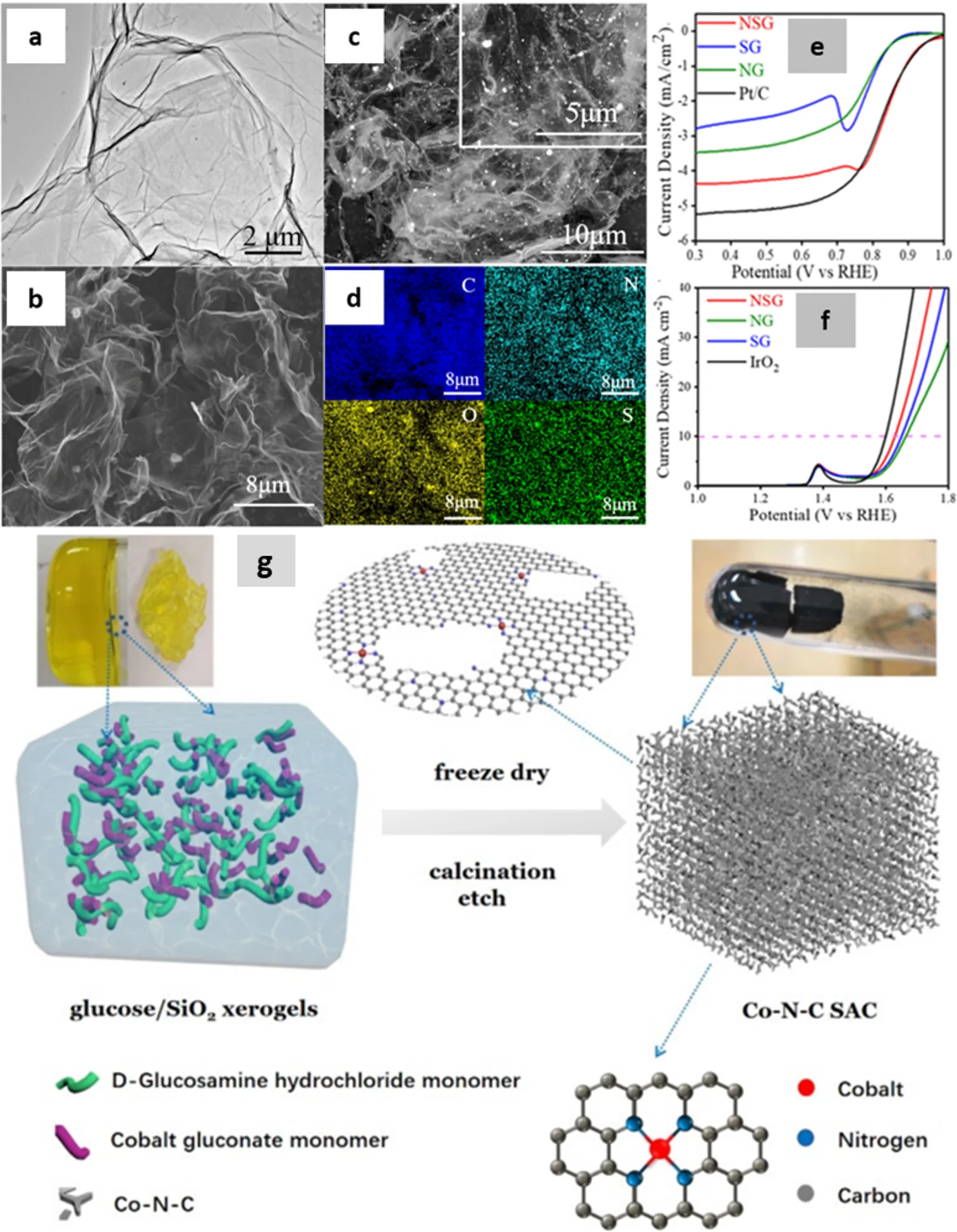
Transmission electron microscopy (TEM) (a), scanning electron microscopy (SEM) (b, c) and mapping (d) of different elements for NSG. (e) ORR linear sweep voltammograms of various catalysts along with Pt/C in O_2_-saturated 0.1 M KOH solution, respectively; (f) OER linear sweep voltammograms of different catalysts with the benchmark IrO_2_ (Figure 3a–f was adapted from [64] (© J. Zhang et al., published by MDPI, Basel, Switzerland, distributed under the terms of the Creative Commons Attribution 4.0 International License, https://creativecommons.org/licenses/by/4.0) (g) Scheme for synthesis of Co–N–C SACs (Figure 3g was adapted from [65] (© L. Wang et al., published by MDPI, Basel, Switzerland, distributed under the terms of the Creative Commons Attribution 4.0 International License, https://creativecommons.org/licenses/by/4.0).

Recently, Yang et al. established a CoFe@(Co_0.5_Fe_0.5_)S N-doped carbon nanotube heterostructured catalyst, which achieved an exceptionally high peak power density of 136.0 mW·cm^−2^, much higher than that of the commercially available Pt/C+ RuO_2_ (102.2 mW·cm^−2^). It conveyed a cycling efficiency of 48.5% after the 800th cycle and operated stably for more than 10,000 min. The strong interfacial coupling within the heterostructure and sulfur-induced electronic modulation, which reduced *OH and *OOH absorption, resulted in the enhanced catalytic performance in both ORR and OER. The catalyst was used in a rechargeable zinc–air battery (ZAB) as a bifunctional oxygen electrocatalyst with very high performance and durability [63]. Another study of heteroatom-doped C, which had N and S doped into graphene oxide, showed superior catalytic properties towards both OER and ORR. The N,S-doped graphene (NSG) was distributed uniformly throughout the surface, which resulted in increased exposure of active sites as shown by the TEM and SEM images in Figure 3a–d. The ORR performance of NSG was compared with that of commercial Pt/C (20%) (Figure 3e), which exhibited onset potential at 0.95 V vs RHE, comparable to the commercial catalyst. Also, a current density of 10 mA·cm^−2^ was achieved at 1.62 V, as shown in the OER linear sweep voltammograms of Figure 3f. This can be attributed to the increased spin density and the facilitated charge transfer due to the mismatch of the outermost orbitals of C and S [64].

The explanation for this is the substitution of larger S atoms into the sp^2^ framework of carbon, which introduces substantial localized structural strain, breaking the symmetry of the conjugated network. The mismatch between the relatively localized C 2p orbitals and the more diffuse S 3p orbitals lowers the charge-transfer resistance and creates fast electron-conduction pathways. The co-doping of N and S into the graphitic network breaks the electroneutrality, optimizing the adsorption free energies of electrochemical intermediates and facilitating rapid faradaic charge transfer across the catalyst–electrolyte interface [60–61]. Wang et al. designed a facile silica xerogel strategy using economic biomass material to synthesise the N-doped carbon network (Co-N-C SAC) shown in Figure 3g. The synthesis process began with the Stöber method to form silica-supported structures, which then underwent freeze-drying. Subsequent pyrolysis under an inert atmosphere enabled the incorporation of nitrogen-doped carbon and cobalt into the silica framework. Finally, acid etching was performed to refine the composite structure. This strategy generated massive mesopore and micropores, which resulted in an outstanding performance of Co–N–C SAC as bifunctional electrocatalyst in alkaline medium in a ZAB [65].

**Carbon-based metal composites.** OER/ORR typically include both electrolyte and oxygen mass transfer and electrical conduction among the active sites and the remaining surface of the electrode. Hence, continuous and efficient transfer between the active sites and the surface ensures better performance of the catalyst. The previous section demonstrated that the creation of defects and heteroatom doping could enhance the performance of the catalyst towards ORR/OER; however, the durability of carbon at high potentials is quite a challenge when it comes to OER. Composites of carbon materials with transition metal compounds are one of the approaches to attain better catalyst activity. Individual components that show good catalytic activity towards ORR or OER can be combined in composites and exhibit strong interfacial chemical interaction and electrical coupling through synergistic effects, thereby resulting into bifunctional electrocatalyst.

**Figure 4:**
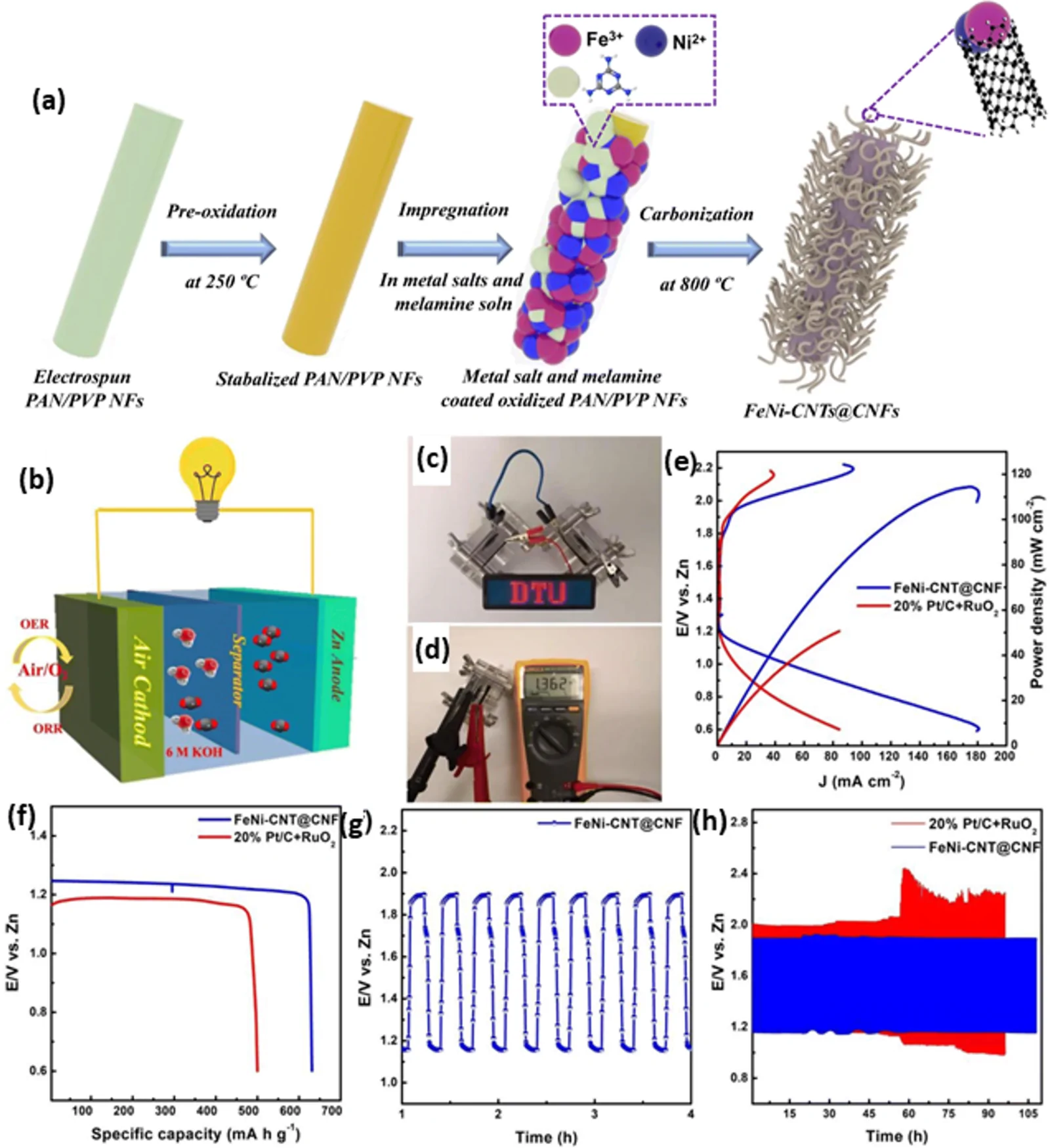
(a) A schematic illustrating the synthesis process of the FeNi-CNT@CNF nanocomposite is presented alongside (b) a visual depiction of the zinc–air battery (ZAB) structure. (c) A photograph of a light-emitting diode (LED, ≈3.0 V) powered by the ZAB demonstrates its operational capability. (d) The open-circuit voltage of the ZAB using the FeNi-CNT@CNF air cathode is shown, followed by (e) galvanodynamic discharge and power density performance. (f) Specific capacity data at a current density of 10 mA·cm^−2^ is also provided, along with (g, h) charge–discharge cycling behaviour of the FeNi-CNT@CNF electrode compared with a 20% Pt/C + RuO_2_-based cathode under identical conditions. (Figure 4 was adapted from [68] (“Growth of carbon nanotubes over carbon nanofibers catalyzed by bimetallic alloy nanoparticles as a bifunctional electrode for Zn–air batteries” © 2023 I. Aziz et al., published by Royal Society of Chemistry, distributed under the terms of the Creative Commons Attribution NonCommercial 3.0 Unported License, https://creativecommons.org/licenses/by-nc/3.0/): This content is not subject to CC BY 4.0).

Strasser and co-workers in 2013 demonstrated a fast microwave-assisted solvothermal one-pot method to synthesise carbon-supported NiFe-LDH and Fe–N–C materials. They were synthesised by repetitive annealing and acid leaching. When these catalysts were mixed, the XRD revealed a two-phase system. The composite of the two individual catalysts, which previously were only showing good activity towards either OER or ORR now exhibited a low overpotential window for ORR/OER in 0.1 M KOH [66]. Alloying of two or more metals causes changes in electron energies, remodelling the surface for better binding spots acting as active sites for electrocatalytic processes [67]. Aziz et al. designed an economical bifunctional electrocatalyst through heat treatment of PAN/PVP precursor nanofibres containing bimetallic nanoparticles and melamine. The schematic representation of the synthesis procedure is shown in Figure 4a. FeNi-CNT@CNF was tested in a ZAB as shown in Figure 4b. To compare the catalyst’s behaviour in the ZAB, 20% Pt/C and RuO_2_ (1:1) was also tested. It was found that both open-circuit voltage and power density for FeNi-CNT@CNF was larger, that is, 1.36 V (Figure 4d) and 118 mW·cm^−2^, respectively, compared to the benchmark catalyst with 50 mW·cm^−2^ (Figure 4e). Also, the specific capacity at 10 mA·cm^−2^ was much higher for FeNi-CNT@CNF (Figure 4f), and the charge/discharge voltage gap at 5 mA·cm^−2^ for 108 h was found to be smaller than that of Pt/C catalyst [68]. Similarly, Yoo and co-workers reported a highly reversible bifunctional electrocatalyst, namely, NiFe alloy nanoparticles confined in CNTs on biaxially stretchable carbon cloth. It showed exceptional mechanical stability and rate performance, which makes it potential candidate for next-generation flexible devices [69].

The use of metal–organic frameworks as templates for synthesizing carbon-based electrocatalytic materials has gained significant momentum lately. This is due to their eco-friendliness, diverse structures, larger surface area, tunability, and high porosity, which ultimately support effective transfer of charges and boost performance [70]. Chen et al. developed a hierarchical architecture composite, that is, Co-NC@LDH, as an excellent bifunctional electrocatalyst. The catalyst contained NiFe-LDH nanosheets which were OER-active, harboured among ORR-active Co-doped CNTs on the surface of ZIF-derived carbon framework sheets. The corresponding potential difference, Δ*E* = 819 mV, is superior to the state of the art. This can be attributed to the LDH nanosheets and CNTs well distributed on the carbon framework while exposing plentiful active sites for both reactions [71]. Another work based on MOFs was done by Li and co-workers, where a honeycomb-like composite was fabricated by pyrolysis of a single-crystal Fe-MOF precursor. The composite showed promising results in a ZAB, and a 2.4 V LED light was powered for more than 24 h by integrating two ZABs [72].

### Transition metal-based materials

Non-noble TMs, like Mn, Fe, Co, Ni, and Zn, are easily available elements, which show activity towards OER and ORR. After modification and optimization, the d orbitals may yield great electron transport efficiency and an excellent electronic structure to the catalyst. Researchers have created numerous superior catalyst materials that contain TMs [73–74]. The most recent developments in TM-based OER/ORR catalysts such as transition metal oxides (TMOs), mix-metal oxides (MMOs), sulfides, carbides, nitrides, and selenides are presented in this section.

The active sites on the catalyst surface for ORR and OER are particularly different, which is the main challenge in this research field [75–76]. Thus, finding the right active sites and controlling the intrinsic activity are essential to obtain high bifunctional performance. In addition, the electrocatalysts’ structural characteristics ought to be adjusted to increase their surface area, speed up electron transfer and mass diffusion, and reveal more active sites that are involved in the catalytic process [77–78]. In order to improve the apparent electrocatalytic performances, the development of active catalysts with advantageous structures and high bifunctional intrinsic activity remains a key research priority.

**Transition metal oxides.** Based on encouraging experimental results, TMs and TMOs are viewed as another type of materials for ORR/OER [79]. To increase the conductivity, metals can be doped in the TMOs structure to create metal-doped TMOs; alternatively, they can be utilized directly as support for electrocatalysts in conjunction with a noble metal [76]. Good ORR performance of TMO-based catalysts is made possible by the presence of lower oxidation states of the TMOs and elements to mix with other active materials. However, under alkaline conditions, TMOs have also demonstrated exceptional efficacy regarding OER [80]. Electrical conductivity, crystal structure (crystallinity and terminating facets), morphology, surface chemistry, and metal valence state all influence TMOs’ electrocatalytic activity towards ORR/OER. TMOs often are semiconductors. Nevertheless, by altering the chemical composition and adding oxygen vacancies, the conductivity can be significantly raised (to the “metal” level) [81].

Manganese oxides. In photosynthesis, oxygen evolution takes place at the oxygen-evolving complex (OEC). Mono-µ-oxo bridges to the fourth Mn ion are discovered to be present in this complex, which is composed of cuban-like Mn_3_CaO_4_ clusters [82]. According to calculations using DFT, every stage of the OER in these materials appears to be nearly thermoneutral, which is necessary for reversible catalysis [83–84]. Therefore, materials based on manganese have the potential to be intriguing bifunctional electrocatalysts for ORR/OER. Furthermore, their low price and excellent stability under alkaline conditions for ORR/OER have garnered a lot of interest lately. Mosa et al. evaluated the effect of the calcination temperature of Cs-doped manganese oxide (CsMnOx), which was produced using a simple soft-templated inverse micelle method. They found that 450 °C was the ideal calcination temperature [85]. Specifically, in the ORR, Cs-MnOx-450 follows the 4e^−^ transfer pathway with a Tafel slope of 69 mV·dec^−1^, which is close to the benchmark Pt/C catalyst. In the OER, Cs-MnOx-450 had a Tafel slope of 100 mV·dec^−1^ and 40 mA·cm^−2^ current density at 2.71 V vs RHE. This excellent catalytic activity was achieved by incorporation of Cs in the Mn_2_O_3_ structure. The presence of Cs increased the crystallinity of the material (via larger interparticle voids in the densely packed Mn_2_O_3_ structure) and stabilized the active Mn^3+^ ions on the surface responsible for ORR and OER.

Shao et al. enhanced Mn_2_O_3_’s catalytic activity by combining two NiO and Mn_2_O_3_ through a straightforward chemical procedure, where carbon dots (CDs) were covalently linked with NiO and Mn_2_O_3_ (NiO–Mn_2_O_3_-CDs). The material exhibited *n* = 3.85 and *j*_d_ ≈ 6 mA·cm^−2^ for ORR, which is close to Pt-based electrocatalysts [86]. NiO–Mn_2_O_3_-CDs yielded 10 mA·cm^−2^ in the OER scenario with 298 mV overpotential and a Tafel slope of 141.14 mV·dec^−1^. The oxygen-containing groups of the CDs are responsible for rapid exposure of the active sites and accelerated charge transfer by covalently bridging the Ni and Mn atoms.

**Figure 5:**
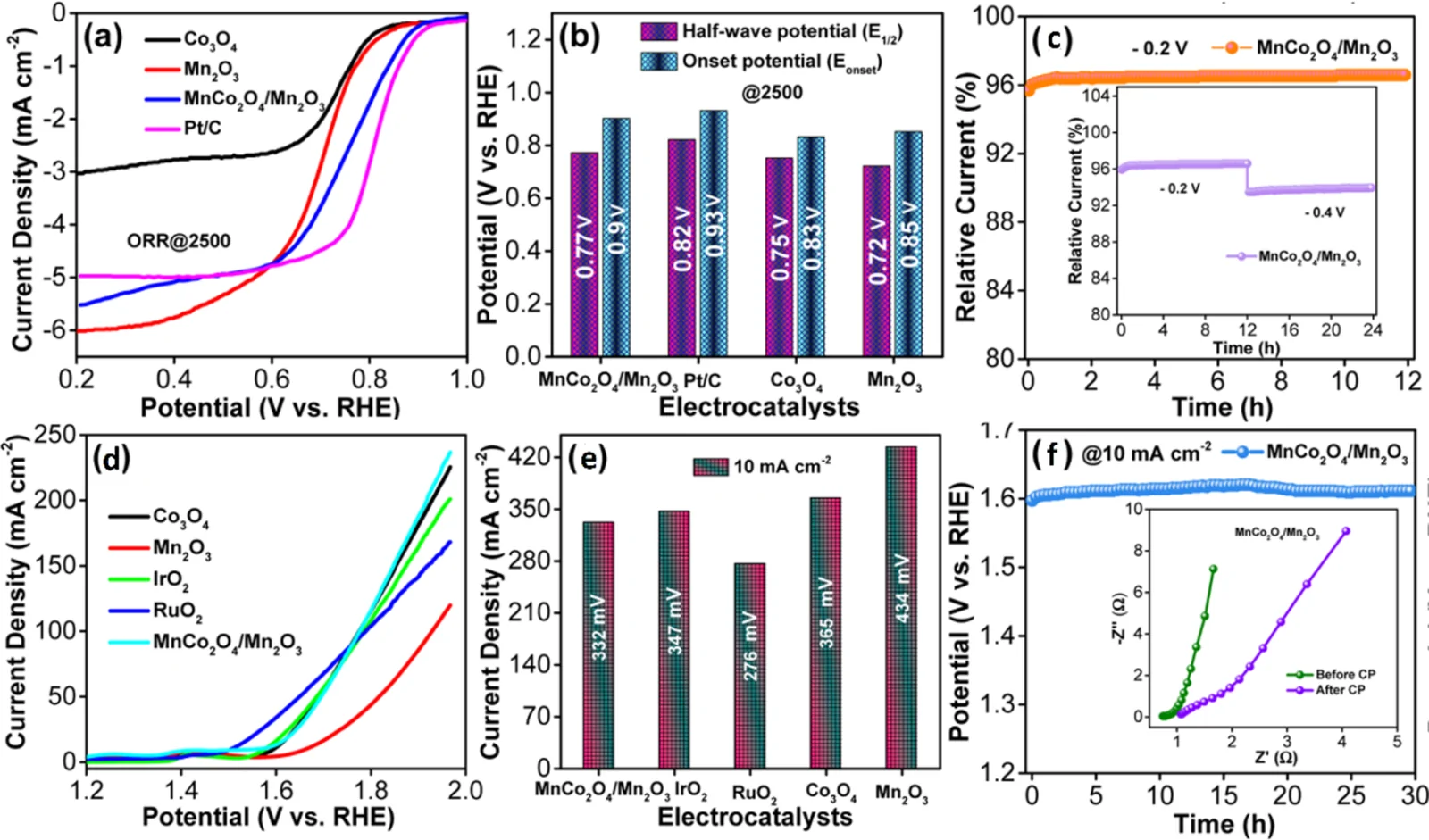
(a) Comparative ORR curves of the MnCo_2_O_4_/Mn_2_O_3_ NR, Mn_2_O_3_, Co_3_O_4_, and Pt/C catalysts at a fixed rotation of 2500 rpm. (b) Onset and half-wave potentials. (c) CA stability test results of the MnCo_2_O_4_/Mn_2_O_3_ NR electrocatalyst at a fixed voltage (−0.2 V) (inset of (c): at different voltages (−0.2 and −0.4 V) in a 0.1 M KOH electrolyte at a fixed rotation of 2500 rpm). (d) Comparative OER curves of the MnCo_2_O_4_/Mn_2_O_3_ NR, Mn_2_O_3_, Co_3_O_4_, IrO_2_, and RuO_2_ catalysts. (e) Overpotentials of the MnCo_2_O_4_/Mn_2_O_3_ NR, Mn_2_O_3_, Co_3_O_4_, IrO_2_, and RuO_2_ catalysts at the current densities of 10 mA·cm^−2^. (f) CP stability test results of the MnCo_2_O_4_/Mn_2_O_3_ NR electrocatalyst at 10 mA·cm^−2^ (inset: EIS plot). (Figure 5 was adapted with permission from [87]. Copyright 2024 American Chemical Society).

Recently, Ayyaluri et al. used a simple one-step hydrothermal process without calcination to create manganese cobalt oxide/manganese oxide (MnCo_2_O_4_/Mn_2_O_3_) nanorods (NRs). The MnCo_2_O_4_/Mn_2_O_3_ NRs showed superior electrocatalytic capabilities for ORR and OER. In comparison to the Pt/C and IrO_2_ catalysts, the MnCo_2_O_4_/Mn_2_O_3_ NR electrocatalyst demonstrated higher durability and high diffusion-limiting current density values. It exhibited remarkable durability across a range of current densities, showing only slight declines in potential. Additionally, it maintained stable chronopotentiometric performance for 30 h under a constant current density of 10 mA·cm^−2^. Besides, compared to the Pt/C//IrO_2_-based ZAB, the MnCo_2_O_4_/Mn_2_O_3_ NR-based ZAB showed a marginally smaller voltage plateau and lower electrochemical impedance values. Notably, the longevity of the MnCo_2_O_4_/Mn_2_O_3_ NR-based ZAB (68 cycles @ about 20.3 h) was superior to that of the Pt/C//IrO_2_-based ZAB (28 cycles @ approximately 8.3 h) (Figure 5 shows the catalytic activity and stability of MnCo_2_O_4_/Mn_2_O_3_ NR compared to other catalysts) [87].

Cobalt oxides. Compared to other metal oxides, cobalt oxides perform reasonably well in ORR and OER, and their potential for enhancement through various synthesis techniques has made them a popular choice for bifunctional catalyst research [88–89]. Even though the precise mechanism of the catalytic active site is unknown, it has been shown that the higher OER activity is related higher levels of Co^3+^ oxidation states and that the ORR activity is related to higher levels of Co^2+^ oxidation states [90]. By tuning the ratio of Co^2+^ and Co^3+^ by different synthesis process, better bifunctional electrocatalysts towards ORR and OER can be developed.

Using a “carbonization–solvothermal–calcination” technique, He et al. prepared a CoO@NC catalyst, which showed excellent bifunctional performance. The CoO@NC-driven zinc–air battery outperforms commercial Pt/C-RuO_2_ catalysts with an impressive peak power density of 112.4 mW·cm^−2^ and operates consistently for over 200 h [91]. Ashok et al. reported a single-step solution combustion method to create porous NiCoO_2_. In the ORR, 3.86 electrons were exchanged and a current density of 3.5 mA·cm^−2^ was achieved [92]. The catalyst was stable up to 24 h for OER with a Tafel slope of 95 mV·dec^−1^. Oxygen vacancies and higher oxidation states are responsible for NiCoO_2_’s higher activity and stability. A dandelion-shaped NiCoO_2_ (NiCo_2_O_4_-DL) catalyst was examined by Liu et al. for alkaline ORR [93]. A 4e^−^ ORR mechanism is indicated by the exchange of 3.91 electrons, with 3.9 mA·cm^−2^ current density. The numerous active Co^3+^ sites, high specific surface area, and three-dimensional porous structure are responsible for the good catalytic performance.

For an effective and long-lasting rechargeable ZAB application, Huang and co-workers recently synthesized a layered cobalt oxide Ba_2_Co_9_O_14_ (BCO) via ball-milling [94]. Ball-milling effectively enhances the material’s surface area, which contributes to a notable improvement in its catalytic efficiency for both ORR and OER under alkaline conditions. Furthermore, the synthesized BCO-based ZAB demonstrated a high specific capacity of 789 mAh g^−1^, exceptional cycling stability exceeding 250 h at a current density of 10 mA·cm^−2^ with more than 1500 charge–discharge cycles, and a significantly elevated peak power density of 166 mW·cm^−2^.

Nickel oxides. Nickel oxide (NiO) is p-type semiconductor, which has long been studied for OER because of its low resistance. It is unstable in acidic medium and can perform better under alkaline conditions [95]. For this reason, researchers are working hard to create and enhance the activity for OER in alkaline medium. Zhang et al. reported a waxberry-shaped cobalt–nickel oxide/S, N-codoped carbon hollow nanocomposites (CoNiO_2_/SNC) as bifunctional catalysts. CoNiO_2_/SNC required an overpotential of 381 mV to maintain 10 mA·cm^−2^ current density for OER. Also, it has a low Tafel slope of 34 mV·dec^−1^. For ORR, CoNiO_2_/SNC has a positive onset potential and a half wave potential of 0.85 and 0.70 V, respectively. Also, CoNiO_2_/SNC has 6.5 mA·cm^−2^ diffusion-limited current density at 1600 rpm for ORR [96]. Faisal and co-workers reported an excellent bifunctional nickel/nickel oxide (Ni/NGr) catalyst supported on nitrogen-doped graphene [97]. For ORR, it has positive onset potential of 0.82 V and 5.0 mA·cm^−2^ current density at 2000 rpm. While for OER, the potential for 10 mA·cm^−2^ current density Ni/NGr is 1.62 V; also, it has a low Tafel slope of 98 mV·dec^−1^ [97].

Zhang et al. recently developed a copper-doped nickel oxide catalyst (Cu*_x_*-NiOSC) via a coprecipitation technique for application in both oxygen reduction and evolution reactions [98]. The Cu_0.2_-NiOSC catalyst displayed a Tafel slope of 57.8 mV·dec^−1^ during the ORR, along with an electron transfer number of approximately 3.6. In the context of the OER, this catalyst achieved a low overpotential of 510 mV at a current density of 10 mA·cm^−2^. Notably, it also exhibited the lowest Δ*E* value of 0.93 V, indicating its superior bifunctional catalytic efficiency.

Regarding their abundant active sites, tunable electronic properties, and structural versatility, transition metal oxide catalysts have become very promising and affordable substitutes for noble-metal catalysts in OER and ORR. Because of the favourable adsorption energies of oxygen intermediates (*OH, *O, *OOH) and the formation of high-valence metal active species during operation, transition metal oxides, especially those based on Ni, Co, Fe, Mn, and their mixed oxides (e.g., spinels and perovskites), exhibit excellent activity for OER. In ORR, oxides like MnO_2_, Co_3_O_4_, and perovskite-type materials exhibit competitive activity in alkaline media through improved electron transfer pathways and better oxygen binding, despite typically having poorer conductivity than carbides or nitrides. Catalytic kinetics and durability are greatly enhanced by techniques including defect engineering (defects like cation or anion vacancies can shift the metal’s d-band centre relative to the Fermi level, which tunes the adsorption energy between catalyst surface and oxygen intermediates), morphology engineering (by controlling the physical shape, size and dimensionality, electrochemically active surface area can be maximized), heterojunction engineering (coupling of two or more distinct materials can modulate the d-band centre and provides dual-function active sites), vacancy engineering (creating metal or oxygen vacancies can change the electronic structure of the catalyst by redistributing electrons to neighbouring atoms, which modulates the conductivity), and composite formation with conductive carbon. Overall, transition metal oxides are moderately active for ORR and very effective for OER, especially in alkaline solutions. The development of good bifunctional oxide catalysts depends on ongoing interface engineering and electronic structure manipulation.

**Transition metal non-oxide materials.** As discussed above, researchers have extensively explored the bifunctional catalytic nature of TMO-based catalysts for OER/ORR. In recent years, researchers are also focussing on transition metal non-oxide electrocatalysts such as metal carbides, metal nitrides, metal sulfides, and metal selenides. They are of relevance to both OER and ORR because to their distinct shapes and surface properties.

An important class of interstitial alloys are transition metal carbides (TMCs), which are formed when carbon atoms dissolve interstitially into parent metal atom lattices. They have unique physicochemical characteristics. They have high melting points, good thermal and electrical conductivities and extreme hardness. Because of these benefits, a variety of metal carbides, including cobalt, tungsten, and iron, have drawn increased interest as OER/ORR electrocatalysts [99–100]. Doping with heteroatoms such as N, B, P, and S is a commonly used approach to boost performance towards the ORR since it exposes active sites and increases conductivity [101–102]. N and B-codoped Co_3_C (Co_3_C-NB) was created by Ma et al. as an oxygen electrode in ZABs. Co_3_C-NB’s charge and discharge polarization curves, together with the power density curves, show that the cathode produces a greater power density than Pt/C + IrO_2_ (36.8 mW·cm^−2^ at 100 mA·cm^−2^), with 45.2 mW·cm^−2^ at 169.4 mA·cm^−2^ [103]. Similarly, Yang et al. reported Fe/Fe_3_C encapsulated in carbon nanotubes, which had a reduced overpotential of 353 mV in OER and bifunctional activity of 0.86 V (vs RHE) for ORR [104].

Transition metal nitrides (TMNs) are also interstitial alloys similar to TMCs. The addition of nitrogen to the interstitial location modifies the parent metal structure. Ionic, covalent, and metallic bonds can develop between metal and nitrogen. TMCs have exceptional stress tolerance, hardness, and high brittleness. Due to strong hybridization between the transition metal d orbitals and nitrogen 2p orbitals, d-band contraction occurs in TMNs. Because of this, TMNs are more conductive than the corresponding metal oxides [105–106]. These characteristics led to increased interest in different transition metal nitride-based electrocatalysts for a range of electrochemical processes and ZAB applications. In 2019, Chen et al. prepared well-dispersed spherical Co_4_N nanocrystals (Co_4_N@NC) using melamine. Its electrochemical performance displays a half wave potential of 0.87 V. Additionally, at a current density of 10 mA·cm^−2^, Co_4_N@NC shows an OER overpotential of 398 mV. With this electrode, the ZAB yields a power density of 98.6 mW·cm^−2^ at 150 mA·cm^−2^ [107]. Wang et al. created Ni_3_FeN/Co,N-CNF by annealing at 400 °C in an NH_3_ environment. Ni_3_FeN/Co,N-CNF outperforms commercial IrO_2_ and Pt/C in terms of overpotential, achieving 270 mV for OER and 0.81 V for ORR, while maintaining the face-centred structure. Furthermore, the Ni_3_FeN/Co,N-CNF cathode had 200 mW·cm^−2^ power density in a ZAB [108]. Yin and co-workers reported NiO/CoN nanowires, which had outstanding catalytic performance because of their many NiO and CoN nanointerfaces with plenty of vacancies, which can increase the catalyst’s stability and catalytic performance. This battery had 945 Wh·kg^−1^ energy density and 79.6 mW·cm^−2^ power density while producing 200 mA·cm^−2^ current density [109].

Table 2 presents a comparison of various parameters related to the performance of the ORR and OER including its performance in ZABs for non-precious bifunctional electrocatalysts described in past few years [110–122].

**Table 2:** Comparison of bifunctional electrocatalysts for ORR and OER performance.

Electrocatalyst	ORR half-wave potential (in 0.1 M KOH)	OER overpotential at 10 mA·cm^−2^ (in 1 M KOH)	Performance in devices such as ZABs in terms of:				

			Specific Capacity (mA·h·g^−1^)	Power density (mW·cm^−2^)	Stability		Ref.

					time (h)	cycles	

NiFe-DG	0.86 V	358 mV	–	148	12	–	[59]
CoFe@(Co_0.5_ Fe_0.5_)S@NCNT	0.88 V	266 mV	–	136.0	166	800	[63]
FeCo-1/NSC	0.82 V	325 mV	–	162.74	150	–	[67]
FeNi-CNT@CNF	0.80 V	310 mV	–	118	108	–	[68]
NiFe–N-CNT-KCC	0.87 V	173 mV	556.54	112.3	90	–	[69]
Co-NC@LDH	0.80 V	389 mV	806	107.8	300	1800	[71]
FeS/Fe_3_C@NS-C-900	–	270 mV	750	90.9	865	1730	[72]
NiO-Mn_2_O_3_-CDs	0.84 V	298 mV	–	287	8.3	50	[86]
Ba_2_Co_9_O_14_	0.71 V	–	789	166	250	1500	[94]
Ni-Co oxide (NC-2)	0.85 V	–	714	–	80	–	[110]
L-Co-NiO@CNT/CC	–	350 mV	756	73.4	960	–	[111]
CoO/NBC	–	–	676.1	127.2	–	400	[112]
NiCo_2_O_4_/MC-HT-O_2_	0.76 V	621 mV	824.86	36.89	–	100	[113]
Co/MnO@NC	0.80 V	354 mV	–	217.7	459	–	[114]
N-MnO_x_-30	0.80 V	–	841.71	181.15	13.8	–	[115]
Mn-Co_3_O_4_@CNTs	0.84 V	356 mV	–	116	425	1200	[116]
(Fe,Co)Se_2_@Fe_1_/NC	0.88 V	266 mV	775	260	280	1500	[117]
c-CoSe_2_-CoN/NC	0.85 V	320 mV	802	118	250	–	[118]
A-Fe_3_C@NO-PC	0.85 V	420 mV	–	136.9	52	300	[119]
Se-doped MOF CoS_2_	0.88 V	290 mV	620.6	–	–	500	[120]
Fe_1.2_(CoNi)_1.8_S_6_ MES	0.81 V	246 mV	–	124	300	–	[121]
Ni–Fe–MoN	0.72 V	228 mV	–	118	20	–	[122]

Recently, Sanchez et al. reported Ni and Co-doped MnS*_x_* (NCMS/NrGO) supported on N-doped reduced graphene which showed catalytic activity towards the ORR with *E*_onset_ of 0.94 V vs RHE with a Tafel slope of 27 mV·dec^−1^. NCMS/NrGO required a potential of 1.59 V vs RHE to maintain 10 mA·cm^−2^ current density for OER. Additionally, it functions as a very effective air cathode for useful zinc–air batteries with long catalyst durability (1560 cycles, approximately 260 h) and high-power densities (124 mW·cm^−2^) [123]. Peng and co-workers synthesized nitrogen-doped hollow carbon spheres (NHCS) loaded in Ni_0.85_Se/Co_0.85_Se with 1:1 mass ratio Ni_0.85_Se/Co_0.85_Se-NHCS-2 for OER and ORR. Ni_0.85_Se/Co_0.85_Se-NHCS-2 demonstrated outstanding ORR activity, with a current density of 4.66 mA·cm^−2^, an onset potential of 0.90 V, and a half-wave potential of 0.77 V. Ni_0.85_Se/Co_0.85_Se-NHCS-2 showed a Tafel slope of 118.3 mV·dec^−1^ and an operational potential of 1.63 V at a current density of 10 mA·cm^−2^ for OER activity, which is somewhat lower than that of RuO_2_ (1.59 V) [124]. A composite of multiwalled carbon nanotubes (MWCNTs) and MnSe (MnSe@MWCNT) was reported by Singh et al. as bifunctional oxygen electrode. The MnSe@MWCNT catalyst composite surpasses state-of-the-art RuO_2_, with a small Tafel slope of 54.76 mV·dec^−1^ with a very low overpotential of 290 mV at 10 mA·cm^−2^ along with *E*_onset_ of 0.94 V for ORR [125].

Because of their numerous active sites, high conductivity, and tunable electronic structures, transition metal carbides, nitrides, sulfides, and selenides have become effective non-noble metal electrocatalysts for the OER and ORR. Although carbides may experience surface oxidation under extreme OER circumstances, metal carbides and nitrides have substantial ORR activity and good bifunctional potential due to their metallic conductivity and advantageous d-band properties. During OER, metal nitrides frequently experience surface reconstruction to produce active oxyhydroxide species, which improves their stability and catalytic activity under alkaline conditions. The true active phases for OER are metal sulfides and selenides, which exhibit rich redox chemistry and easy surface transformation into metal (oxy)hydroxides. Selenides typically have better conductivity and charge transfer than sulfides, which leads to superior OER and competitive ORR performance. Overall, carbides and nitrides are particularly promising for ORR due to their conductivity and electronic structure, while sulfides and selenides excel in OER through in situ surface reconstruction; rational design strategies such as heterostructure engineering, defect creation, and heteroatom doping are key to achieving highly efficient and durable bifunctional ORR/OER catalysts.

## Conclusion

The development of OER/ORR non-precious bifunctional electrocatalysts for electrochemical conversion and storage devices greatly simplifies systems and cuts down the cost regarding commercialisation. However, for bifunctional electrocatalysts, the performance indicators for both reactions need to be taken into account equally, which is challenging. In this contribution, carbon- and transition metal-based materials as bifunctional oxygen electrocatalysts have been reviewed. The carbon-based catalysts have advantages, that is, abundance in nature, structural diversity, environmental compatibility, and high conductivity. However, only a very small number of carbon-based materials without any inclusion of transition metals have been tested for practical application. Many transition metal/carbon composites have been reported as electrocatalysts to show better activity than commercial ones (Pt/Ru/Ir based) in alkaline medium and have also been tested in devices like ZABs regarding stability/durability in practical settings. The activity of non-precious electrocatalysts in acidic medium has quite a lot of room of improvement. The catalysts’ activities towards ORR/OER in acidic medium can be then compared with one of the studies done by Ruck et al. who deposited Ir on Pt black nanoparticles to obtain a bifunctional catalyst for acidic media [126]. Also, durability of the carbon-based catalyst at higher potentials (>1.2V vs RHE) for OER is quite a challenge as they are susceptible to corrosion. There is a lack of precise control of structure, composition, and active sites, which includes controlling the localization of the dopants in order to increase the activity of the electrocatalysts. Another vital parameter that is mostly ignored is catalyst degradation (including dissolution, oxidation, reconstruction, and carbon corrosion), which is a critical bottleneck for the practical deployment of bifunctional oxygen electrodes, especially under the alternating reducing and oxidizing conditions of ORR and OER. Additionally, study regarding bifunctionality will be more meaningful with in situ characterization and theoretical calculations to have a better understanding of the mechanistic pathway to determine the real active sites. Above all, since these catalysts are developed in laboratories, to check their compatibility with commercial scenarios, practical devices such as fuel cells and metal air batteries must be built. Some of the reported studies have carried out tests in ZAB-like devices, and this will be the ultimate requirement to verify whether a catalyst will be scalable or not for industrial levels.

In conclusion, this review intends to give an insight to the recent advances of non-precious metal bifunctional oxygen electrocatalysts. There is no doubt that there are advances in this field, and there are various design strategies to improve the activity of catalysts to a point where it can compete the state-of-the-art Pt/C, RuO_2_, and IrO_2_; however, further improvements are needed for commercial applications. We believe, the review could provide some useful insights for future progress in carbon or transition metal-based bifunctional oxygen electrocatalysts.

## Data Availability

Data sharing is not applicable as no new data was generated or analyzed in this study.
